# Quarantined Patients without Food

**Published:** 1898-05

**Authors:** 


### Quarantined Patients Without Food.

At Middleboro, Ky., forty cases of variola and twenty-nine suspects are quarantined in the pest-house, and there are no funds to pay for their care. Dr. McCormack, chief inspector of the State Board of Health, says the State has no funds to be used for this purpose, the county refuses to make an appropriation, and the city is bankrupt. Surgeon Wertenbaker, of the Marine Hospital Service, who was sent to investigate the situation, is anxious and willing to render Federal assistance, but can only do so on invitation of the State Board of Health. Dr. McCormack vigorously opposes Federal intervention, and states that if the county does not render the necessary aid, he will withdraw and release the patients. Meanwhile the latter have been without food for two days and threaten to make their escape.—*American Practitioner and News.*

